# Influence of Season and Liquid Storage at 16 °C on Beni Arouss Bucks’ Semen Quality

**DOI:** 10.3390/ani10111986

**Published:** 2020-10-29

**Authors:** Sara El Kadili, Nathalie Kirschvink, Marianne Raes, Jean Loup Bister, Bouchaib Archa, Ahmed Douaik, Mouad Chentouf

**Affiliations:** 1 Department of Veterinary Medicine, Integrated Veterinary Research Unit, Namur Research Institute for Life Sciences (NARILIS), Faculty of Sciences, University of Namur, Rue de Bruxelles 61, B-5000 Namur, Belgium; marianne.raes@unamur.be (M.R.); jeanloup.bister@gmail.com (J.L.B.); 2 Department of Animal Production, Ecole Nationale d’Agriculture de Meknès, Route Haj Kaddour, BP. S/40, 50001 Meknes, Morocco; barcha@enameknes.ac.ma; 3 Institut National de la Recherche Agronomique, Regional Center of Rabat, PB 6570, 10101 Rabat, Morocco; ahmed_douaik@yahoo.com; 4 Institut National de la Recherche Agronomique, Regional Center of Tangier, Bd Sidi Mohamed Ben abdellah78, 90010 Tangier, Morocco; mouad.chentouf@gmail.com

**Keywords:** Beni Arouss buck, season, liquid storage, motility, viability, normal morphology

## Abstract

**Simple Summary:**

Goat production plays an important economic and social role in Northern Moroccan farming. Beni Arouss is an autochthonous North Moroccan goat breed. The use of artificial insemination can largely contribute to optimised preservation and dissemination of valuable traits of this breed and lead to improved productivity. During liquid storage, the stability of semen good quality remains crucial in order to provide greater flexibility between the artificial insemination center and farms where the insemination of does needs to be performed. The study aims to assess the effect of storage and season of the year on fresh semen of Beni Arouss goats. Every month, ejaculates were collected from bucks and were extended and stored at 16 °C for 24 h. Semen motility, viability and normal morphology were assessed at 0, 4, 8 and 24 h after collection. As expected, these parameters showed a significant reduction within 24 h of storage and during all seasons. However, semen collected in summer maintained a better quality after 24 h of storage at 16 °C than semen collected during the other periods. Therefore, the storage ability of Beni Arouss bucks’ semen stored at 16 °C was higher during the summer.

**Abstract:**

The study aimed at determining the effect of storage and season on fresh semen of Beni Arouss goats. Ejaculates were collected at monthly intervals from seven mature bucks and were extended at a final concentration of 800 × 10^6^ spermatozoa. ml^-1^ and stored at 16 °C for 24 h. Semen motility, viability and normal morphology were assessed at 0, 4, 8 and 24 h after collection. Motility and normal morphology parameters were recorded using computer-assisted sperm analysis (CASA) and viability was analyzed using eosin–nigrosin staining. As expected, motility, viability and normal morphology parameters showed a significant reduction within 24 h of storage and during all seasons (*p* < 0.05). However, semen collected in summer maintained a better quality after 24 h of storage at 16 °C than semen collected during the other periods (*p* < 0.05). In conclusion, the storage ability of Beni Arouss bucks’ semen stored at 16 °C was significantly higher during the summer.

## 1. Introduction

In the North of Morocco, goat production plays an important social and economic role and represents a live-hood base for 70% of the rural population [1]. However, low productivity limits the producer’s income [2]. This has led farmers to replace local goats with higher yielding foreign breeds or to perform crossings between local and foreign breeds. This large-scale dissemination of imported dairy goat breeds led to the erosion of indigenous goat genetic resources. In 2007, the food and agriculture organization (FAO) launched the global plan of action for animal genetic resources to strive against the loss of animal genetic diversity and to preserve the zoogenetic resources.

Beni Arouss is an autochthonous North Moroccan goat breed, recently recognized by the Moroccan Ministry of Agriculture (official Journal of Kingdom of Morocco; No. 6430; 01/2016), whose name is derived from the geographical location. This breed is characterized by good milk production performance and an excellent adaptation to local conditions and resistance to pests and diseases [3]. A breeding program aiming at preservation and improvement of the production potential of Beni Arouss goats is ongoing. The implementation of an artificial insemination (AI) center to support this program is planned by the government. The use of AI should largely contribute to the optimised preservation and dissemination of valuable traits and should lead to improved productivity of North Moroccan goats.

In recent years, AI with fresh semen has become a common technique in goats [4,5,6]. However, the stability of good quality semen during liquid storage remains crucial in order to provide greater flexibility between the AI center and farms where the insemination of does needs to be performed.

An earlier study of our group showed that the quality of Beni Arouss buck semen is influenced by the season of the year [7]. The influence of liquid storage [8] and cryopreservation [9,10,11] on semen quality has been shown to vary in function of seasons. To our knowledge, there are no studies investigating the influence of season on liquid storage of North Moroccan buck sperm. Therefore, the present study aims to evaluate the effect of storage at 16 °C and of the season on the quality of North Moroccan buck semen.

## 2. Materials and Methods 

### 2.1. Animal Management and Semen Collection

The study was conducted from March 2015 to February 2016 at the experimental station of INRA, Regional Center of Tangier, located at the North of Morocco (35°44′ N, 5°54′ O). Seven sexually mature Beni Arouss bucks aged between 5 and 8 years were investigated for one year. They underwent the same management and were maintained indoors in individual pens and under natural photoperiod with uniform feeding. Diet consisted of oat hay and concentrate feed mixture distributed according to the recommended requirements of INRA [12]. Water was available ad libitum. 

Semen was collected monthly by use of an artificial vagina over a 12-months period and stored for 24 h at 16 °C during the four seasons of the year: spring (from 21 March to 21 June), summer (from 22 June to 21 September), autumn (from 22 September to 21 December) and winter (from 22 December to 22 March). At each collection, only the first ejaculate from each buck was evaluated and stored.

### 2.2. Semen Quality Assessment and Processing

Immediately after collection, semen was immersed in a water bath at 37 °C. A predilution (v:v) using a pre-warmed lipid-free synthetic extender elaborated at laboratory level (pH = 7.2 and osmotic pressure = 320 mOsm/Kg) was performed to preserve spermatozoa. Sperm concentration was determined by manual counting with a Bürker haemocytometer. The semen sample was further extended to reach a final concentration of 800 × 10^6^ spermatozoa per mL. Given that semen concentration changed between animals and collections, the volume of extender needed varied to a certain extent (around 20%, which can be considered as negligible with regard to semen quality [13]).

After evaluating semen quality at dilution (time 0), each semen sample was divided into three vials (for T4, T8 and T24) and was progressively cooled to 16 °C by placing a 37 °C-warm water bath containing the vials in a refrigerator. As soon as the temperature of 16 °C was reached, the vials were kept at 16 °C. Sperm motility, viability and normal morphology were determined after 4, 8 and 24 h of storage. Sperm motility was assessed using a computer-assisted sperm analysis (CASA) system (ISAS, Proiser R + D SL, Spain) as described by El Kadili et al. [7]. Motility parameters measured included total motility (TM, %), progressive motility (PM, %), rapidly progressive motile spermatozoa (rapid spermatozoa RAPID, %), velocity according to the actual path (curvilinear velocity VCL, µm/s), velocity according to the straight path (straight-line velocity VSL, µm/s) and velocity according to the smoothed path (average path velocity VAP, µm/s). Viability was determined using the eosin–nigrosin staining. Smears were prepared by mixing 5 µL of eosin, 5 µL of nigrosin and 5 µL of diluted semen (concentration of 800 × 10^6^ spermatozoa/mL) on a warm slide, allowed to react for 30 s and immediately spread with another slide. Viability was assessed by counting 200 cells under a bright-field microscope (60×) and the unstained spermatozoa were considered alive [14]. Sperm morphology was evaluated using the Diff-Quik staining Kit (Microptic Automatic diagnostic system, Barcelona, Spain). Slides were prepared by smearing 3 µL of the semen sample, from the final dilution of 800 × 10^6^ spermatozoa/mL. Smears were prepared stained and examined as described by El Kadili et al. [7]. At each time point, one sample per buck was heated to 37 °C by the use of a heated water bath. The semen analyses were performed within 15 min and the analysed samples were discarded.

### 2.3. Statistical Analysis

Data analysis was performed using PROC MIXED of SAS 9.0 software (SAS Inst. Inc., Cary, NC, USA). Mean values and standard deviations were analyzed in function of the season of the year (spring, summer, autumn and winter) and storage duration (T0, T4, T8 and T24 h). Changes in semen quality parameters after 24 h were further calculated for each buck as a percentage of T0 values (considered as 100%). An ANOVA model for repeated measures was used for each parameter of sperm quality. Prior to analysis, variables expressed as percentages were transformed to arcsine. The statistical model included the fixed effects of season and storage duration. The buck’s identity was treated as a repeated effect. The first-order autoregressive covariance structure was selected, based on the Schwarz Bayesian criterion [15]. The least-squares mean for seasons was compared using the PDIFF option. Data were expressed as mean ± SD and the level of significance was set at *p* < 0.05.

## 3. Results

**Figure 1:**
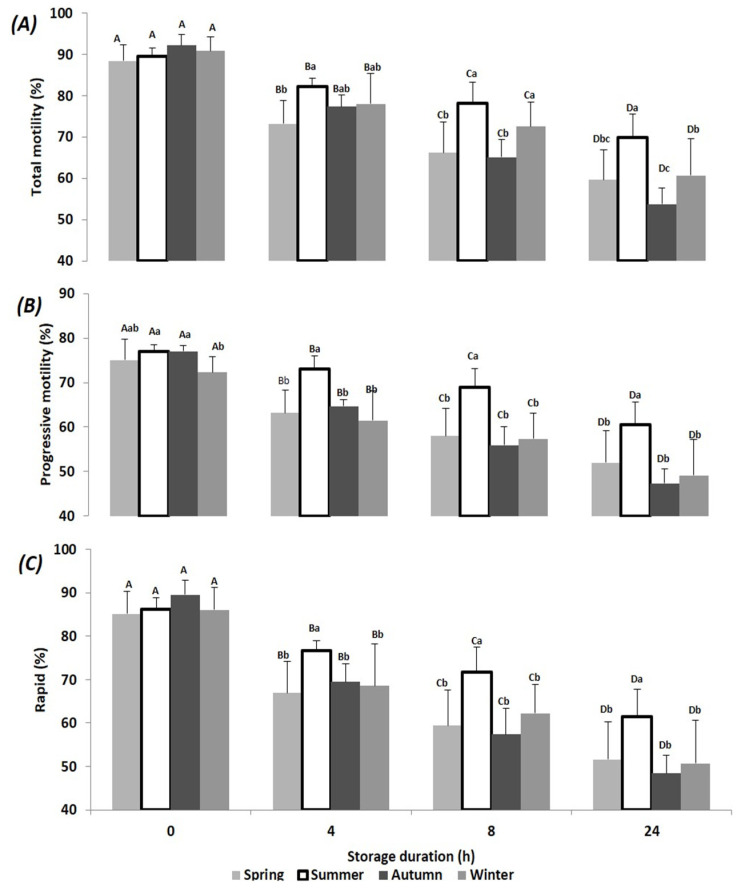
Mean (±SD) of Beni Arouss bucks’ sperm total motility (**A**), progressive motility (**B**) and rapidly progressive motile spermatozoa (**C**) after 0, 4, 8 and 24 h of storage at 16°C and during the four seasons of the year. ^A, B, C, D^: different capital letters indicate a significant effect of storage duration within the same season (*p* < 0.05). ^a, b, c^: different lower-case letters indicate a significant effect of season at the same storage duration (*p* < 0.05).

**Figure 2:**
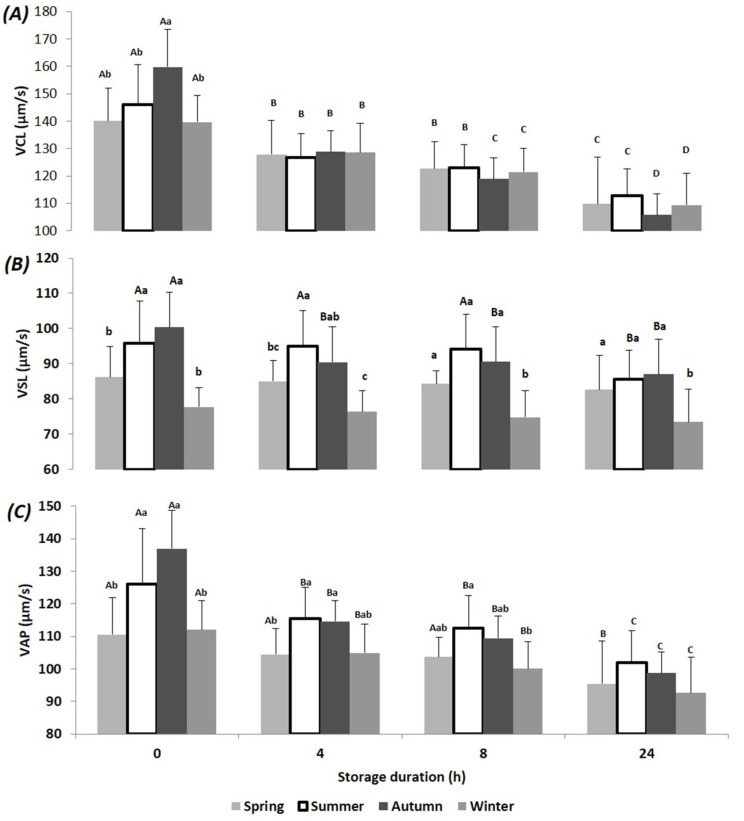
Mean (±SD) of Beni Arouss bucks’ sperm velocities: curvilinear velocity (**A**), straight-line velocity (**B**) and (**C**) average path velocity after 0, 4, 8 and 24 h of storage at 16 °C and during the four seasons of the year. ^A, B, C, D^: different capital letters indicate a significant effect of storage duration within the same season (*p* < 0.05). ^a, b, c^: different lower-case letters indicate a significant effect of season at the same storage duration (*p* < 0.05).

**Figure 3:**
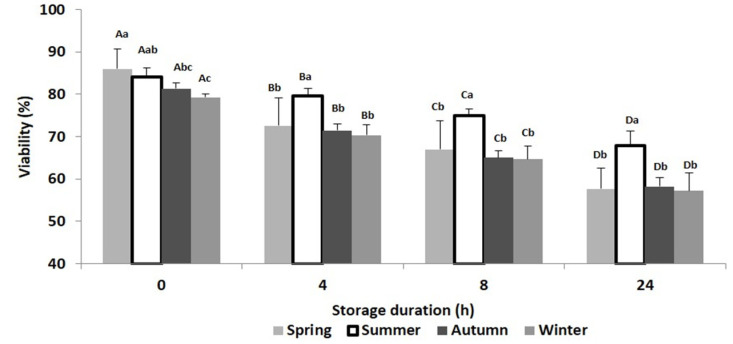
Mean (±SD) of Beni Arouss bucks’ sperm viability after 0, 4, 8 and 24 h of storage at 16 °C and during the four seasons of the year. ^A, B, C, D^: different capital letters indicate a significant effect of storage duration within the same season (*p* < 0.05). ^a, b, c^: different lower-case letters indicate a significant effect of season at the same storage duration (*p* < 0.05).

**Figure 4:**
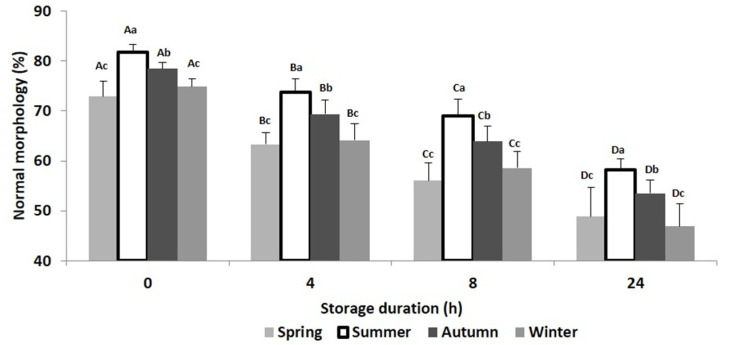
Mean (±SD) of Beni Arouss bucks’ sperm normal morphology after 0, 4, 8 and 24 h of storage at 16 °C and during the four seasons of the year. ^A, B, C, D^: different capital letters indicate a significant effect of storage duration within the same season (*p* < 0.05). ^a, b, c^: different lower-case letters indicate a significant effect of season at the same storage duration (*p* < 0.05).

In the present study, a total of 21 ejaculates were collected from the first ejaculation of seven bucks in each season. The results of semen motility, viability and normal morphology at T0, T4, T8 and T24 and at each season are presented in Figure 1, Figure 2, Figure 3 and Figure 4. Relative changes between T0 and T24 per season are shown in Table 1. Significant effects of storage duration as well as of seasons were found for most variables and are presented below. 

As analyzed and discussed elsewhere in detail [7], the season did not significantly affect rapidly progressive motile spermatozoa in fresh and uncooled semen (T0) (*p* > 0.05). The highest velocity results were recorded during summer and autumn (*p* < 0.05), whereas the lowest progressive motility was recorded during winter (*p* < 0.05). The rate of living spermatozoa was maximum in spring and summer and lowest in winter (*p* < 0.05). The highest semen concentration and percentage of normal sperm were recorded during summer followed by autumn while the lowest counts were observed during winter and spring (*p* < 0.05). 

After 24 h of storage, all parameters of motility, viability and morphology remained at their highest level in summer (*p* < 0.05), the quality loss being lowest during this season. While the best values of motility at T0 were recorded in autumn, the most important quality loss during storage was also recorded at this time point (Table 1).

More precisely, total and progressive motility as well as the percentage of rapid spermatozoa underwent a significant reduction over time during all seasons. Interestingly, T0 values of motility tended to be highest in autumn but underwent the most important storage-related loss during this season, whereas the reduction was lowest in summer (Figure 1 and Table 1). 

Regarding variables characterizing spermatozoa speed, a slight but significant storage effect was recorded for VCL at each time point and for VAP after 24 h. As observed for motility variables, the less important speed changes were recorded in summer despite the fact that T0 values tended to be highest in autumn (Figure 2). 

Semen viability also underwent a time-related significant decrease at each season, but as for the preceding variables, storage had its lowest impact during summer (Figure 3, Table 1). 

Finally, storage-related changes of morphology occurred, as well as a significant decrease in normal spermatozoa. The impact of season was significant: lowest changes were recorded during summer, whereas the highest changes were recorded during winter (Figure 4). 

By considering the amplitude of time-related changes of most recorded variables, progressive and significant differences recorded between T0 and T4, T4 and T8 and T8 and T24 suggested that semen quality loss occurred progressively and increased gradually over time. 

## 4. Discussion

This study is the first to describe storage- and season-related quality change of Beni Arouss bucks’ semen kept for 24 h at 16 °C in a synthetic extender. Sperm motility evaluation was performed by CASA, the system approved for reproducible and accurate assessment of sperm motility parameters [16,17,18]. Several studies demonstrated that motility and velocity parameters generated by CASA could be of use to evaluate semen quality and predict fertility [19,20,21]. In this context, significant positive correlations between different velocity parameters like VCL, VSL and VAP and the percentage of fertilization were reported. Indeed, sperm with decreasing motility parameters seems to undergo a gradual loss of energy needed to produce an adequate straight and progressive movement that is required for fertilization [22]. 

As described elsewhere, an influence of season on Beni Arouss bucks’ libido, and semen characteristics has been evidenced [7]. Briefly, this previous investigation shows that Beni Arouss bucks’ reproduction capacity is maximal in autumn, which corresponds to the natural reproduction period of this breed [23]. The present study focuses on the impact of the storage of fresh semen in the function of the season. Results of semen preservation at 16 °C in a synthetic extender for 24 h indicate that the semen quality dropped progressively between 0 and 24 h of storage. These observations are in line with previous studies showing that during liquid storage, all variables evaluating motility, viability and normal morphology undergo a significant decrease, regardless of the extender in use, dilution rate, temperature and storage conditions [24,25]. This storage-related quality loss was further subjected to seasonal changes. Although the available literature describing seasonal changes of semen quality focuses mainly on thawed semen, it appears that components of seminal plasma and/or of the extender also impact conservation ability [9,26,27,28].

In the present study, all parameters of sperm quality except velocities remained after 24 h of storage at a higher level in summer: quality loss due to storage at 16 °C ranged from 19 to 29%, whereas it ranged from 28 to 46% in autumn. Considering these results, it can be hypothesized that the capacity of spermatozoa to withstand liquid storage was only partially dependent on the quality of semen before storage, but that seasonal changes of seminal components might impact on quality during storage. 

Seminal plasma is a mixture of cauda-epididymis and various male accessory glands secretions discharged during ejaculation [29]. Described as a nutritive and protective medium for suspended sperm cells, the seminal plasma ensures significant roles for sperm metabolism, sperm function and survival, as well as for the control of sperm motility and preservation ability [30,31]. Several studies underlined that ram seminal plasma contains numerous predominant protein families including the BSPs (Binder of sperm proteins) and sperm adhesins [32,33,34]. Some authors have reported that BSPs with an apparent molecular weight above 3 kDa and especially proteins P14 (phosphoprotein) and P20 (glycoprotein) from seminal plasma can prevent or reverse membrane damage induced by cold shock in ram semen, thereby revealing the essential role of seminal plasma in the stabilization of the sperm membrane [35,36,37]. Considering the increase in reproductive activity in Beni Arouss bucks in summer after the seasonal anoestrus known in does [1], concentrations of seminal plasma proteins would be expected to increase and therefore the capacity of non-washed sperm to withstand liquid storage could be improved. It remains, however, unclear why this potential protective effect would not persist in autumn, during the physiological reproduction period of this breed. Given that protective [38,39,40,41] as well as deleterious effects [42,43] of BSPs have been reported in several species, their role on cooled bucks’ semen remains to be established.

Moreover, during semen storage, changes in sperm metabolic performance are observed. During liquid storage, spermatozoa lose their ability to generate ATP through mitochondrial respiration due to mitochondrial ageing [44]. A decrease in mitochondrial activity and loss of ATP during liquid storage is known to have detrimental effects on sperm motility and consequently on sperm function [45,46]. Moreover, the susceptibility of mitochondria to oxidative damage during storage seems to be increased. Spermatozoa are protected from oxidative stress by antioxidants enzymes, mainly by superoxide dismutase (SOD), catalase (CAT) and glutathione peroxidase (GPx) [47,48]. Thus, the enzyme levels of seminal plasma are very important for sperm metabolism as well as sperm function [49]. Antioxidants are further involved in the prevention of cold shock [50] and seasonal changes of the antioxidant capacity of seminal plasma are reported in many species [50,51,52]. The relation between metabolic activity of semen, associated antioxidant consumption and sperm quality remains however unclear. In goats, there are no studies investigating the effect of season on antioxidants enzymes and the association of these enzymes with seminal traits of fresh and cooled semen. It can only be hypothesized that the reduced resistance of autumn-collected semen to liquid storage at 16 °C may be related to and enhanced by increased consumption of antioxidant enzymes in spermatozoa displaying a maximal metabolic activity. 

Lastly, the goat semen presents characteristics that differentiate it from other species, the most important is the presence of lipases secreted by the accessory bulbourethral gland. These enzymes also called EYCE and BUSgp60 are responsible for the reduction of motility and viability of semen cooled or frozen in extenders containing egg yolk or milk [4,53,54]. EYCE that has a phospholipase A1 activity hydrolyzes egg yolk lecithin into fatty acids and lysolecithin [55] and BUSgp60 hydrolyzes triglycerides in skimmed milk into fatty acids (acid oleic) [56]. These hydrolyses make the sperm membranes more fusogenic thereby inducing the acrosome reaction and chromatin condensation, which is toxic to sperm [57,58]. Therefore, the removal of seminal plasma by semen washing is recommended, especially during the non-breeding season when the negative impact of seminal plasma seems maximal (increase in lipase release by the bulbourethral glands) [59,60]. In the current study, the activity of lipases was not expected to exert any negative effect on the quality of extended buck sperm without seminal removal because a lipid-free extender was used. 

The final interesting point is the expected fertilization capacity of Beni Arouss semen stored at 16 °C. In terms of semen quality, after 24 h of storage, our results are similar to those recorded in rams by Ohara et al. [24]. The authors further assessed the gestation rate obtained after cervical IA with fresh semen and semen stored for 24 h at 5 °C in ewes and reported respectively 63% and 54%. Even if it is indispensable to perform IA tests in Beni Arouss goats, the present results suggest that semen stored at 16 °C for 24 h is useable for IA.

## 5. Conclusions

In conclusion, the present study showed that the season significantly affects the characteristics of Beni Arouss buck unwashed semen stored at 16 °C. The lowest quality loss after 4, 8 and 24 h of storage was recorded during summer, whereas the highest quality loss occurred during autumn. Although untested in the present investigation, it might be expected that semen of Beni Arouss bucks leads to satisfying fertility when used within 24 h of storage at 16 °C. 

## Figures and Tables

**Table 1:** Motility, viability and morphology loss (in %) of Beni Arouss buck semen after 24 h of storage at 16 °C.

Traits	Season			
	Spring	Summer	Autumn	Winter
Total motility	32.6 ± 7 ^a^	21.1 ± 5 ^b^	41.7 ± 4 ^a^	33.3 ± 8 ^a^
Progressive motility	30.7 ± 9 ^a,b^	21.4 ± 5 ^b^	38.6 ± 4 ^a^	32.3 ± 9 ^a,b^
Rapid	39.4 ± 9 ^a,b^	28.8 ± 6 ^b^	45.8 ± 6 ^a^	41.2 ± 9 ^a^
VCL	21.6 ± 9 ^b^	22.4 ± 6 ^a,b^	33.4 ± 7 ^a^	21.7 ± 5 ^b^
VSL *	3.6 ± 11	10.5 ± 6	12.5 ± 10	5.5 ± 9
VAP	13.5 ± 11	18.5 ± 7	27.5 ± 8	17.3 ± 7
Viability	33 ± 3 ^a^	19.2 ± 4 ^b^	28.4 ± 3 ^a^	27.9 ± 4 ^a^
Normal morphology	32.8 ± 9	28.8 ± 2	31.8 ± 3	37.4 ± 5

Data are means ± SD. ^a, b^: Means with different superscript letters in the same row are significantly different (*p* < 0.05). * Variations of straight-line velocity (VSL) by season and storage time appear as inconsistent and are not analysed statistically.
